# Effects of Cultured *Cordycep militaris* on Sexual Performance and Erectile Function in Streptozotocin-Induced Diabetic Male Rats

**DOI:** 10.1155/2020/4198397

**Published:** 2020-11-13

**Authors:** Sureena Pohsa, Wanthanee Hanchang, Nattapong Singpoonga, Peerasak Chaiprasart, Pornnarin Taepavarapruk

**Affiliations:** ^1^Department of Physiology, Faculty of Medical Sciences, Naresuan University, Phitsanulok 65000, Thailand; ^2^Faculty of Science and Technology, Nakhon Sawan Rajabhat University, Nakhon Sawan 60000, Thailand; ^3^Department of Agricultural Science, Faculty of Agriculture, Natural Resources and Environment, Naresuan University, Phitsanulok 65000, Thailand

## Abstract

*Cordyceps militaris* (CM), a valuable edible and medicinal fungus, has been used as traditional medicine to treat health conditions, as well as hyposexuality in Asian societies for over a century. Due to the high demand, several artificial cultivation methods have been developed for their biological activities. In this study, CM was cultured on medium that contained white rice and silkworm pupae, and the levels of cordycepin and adenosine, as well as its aphrodisiac effects in diabetes-induced erectile dysfunction (DIED), were evaluated. Diabetic rats were induced by streptozotocin (STZ) injection and administered orally with CM (0.1, 0.5, and 1.0 g/kg BW/day) for 3 weeks. Diabetic rats in negative and positive control groups received vehicle and sildenafil citrate (5 mg/kg), respectively. Results showed the changes in mating behaviour in which mount latency and intromission latency were significantly increased in diabetic rats, compared with the normal control group. Diabetic rats also showed a significant reduction in intracavernosal pressure (ICP) response to cavernous nerve stimulation, sperm count, testosterone level, penile nitric oxide synthase (NOS), and testicular superoxide dismutase (SOD) activities, when compared to the normal control group. Administration of CM (0.1, 0.5, and 1.0 g/kg BW/day) reversed the effects of diabetes on the mating behaviour, and the ICP responses to electrical stimulation. Moreover, the levels of penile NOS, testicular SOD activities, testosterone, and sperm count were significantly increased, and testicular malondialdehyde (MDA) levels were significantly decreased in these treated diabetic rats. Diabetic rats treated with sildenafil showed a significant induction in intromission frequency and NOS and SOD activities, as well as a marked increase in ICP responses. These results suggest that CCM exerts its aphrodisiac effect, possibly through activating testosterone production and suppressing oxidative stress to enhance erectile function in diabetic rats.

## 1. Introduction

Erectile dysfunction or ED has been signified as an inability of the male to achieve a penile erection, as part of the overall multifaceted process of male sexual function [1]. ED status can arise in adult men of all ages, as its prevalence and incidence are associated with aging. The prevalence of ED in young men has been estimated to be as high as 30%, in which diabetes mellitus (DM) either type 1 or type 2 has a well-established and strong association with ED [2, 3]. The pathophysiology of diabetes-induced erectile dysfunction (DIED) is multifactorial and several mechanisms of ED have been proposed in diabetic patients, including increased oxygen free radicals and impaired nitric oxide (NO) synthesis [4]. The chronic hyperglycemia can lead to endothelial dysfunction, which is manifested as the decreased bioavailability of NO, resulting in insufficient relaxation of vascular smooth muscle of the corpora cavernosal [5]. The current first-line therapy for diabetic ED is phosphodiesterase type 5 inhibitors (PDE5Is), such as sildenafil (Viagra®), tadalafil (Cialis®), and vardenafil (Levitra®). However, PDE5-Is has been shown to have some adverse effects, i.e., headache, abnormal vision, dyspepsia, flushing, nasal congestion, and back pain, which may impact negatively on patient's lifestyle [6].

Alternative approaches, such as herbal medicine, have been adopted for sexual improvement for centuries. To date, many plants have been reported to possess aphrodisiac potential, and their effects on sexual behaviour have been validated [7]. In Asian countries, herbal supplements derived from Cordyceps species have been traditionally used as prosexual agent, one of which is *Cordyceps militaris* (CM), a valuable medicinal mushroom in the family Clavicipitaceae [8]. Nowadays, instead of harvesting from natural resources, CM has been cultivated using the artificial culture medium and similar bioactive contents and medicinal potential as wild Cordyceps has been reported [9]. Due to the growing demand, a number of culture techniques and the media formula to support growth of CM have been developed, and many bioactive ingredients have been isolated, such as adenosine, cordycepin, D-mannitol, polysaccharides, nucleosides, amino acid, essential oils, ergosterol peroxides, and xanthophylls [9–11]. Several scientific evidences related to the mechanisms and efficacy of this fungus, such as anticancer, antihypertensive, antioxidant, antiapoptotic, and hypoglycemic effects, have been reported [9, 10, 12–14]. Its positive effects in sexual function and testicular function have also been elucidated in young male rats [15], middle-aged rats [16], and aged male rats [17]. However, research on its bioactivity as prosexual agent in DIED is still scarce. The objective of the present study was to ascertain if CCM had aphrodisiac activity in STZ-induced diabetic rats and to elucidate the underlying mechanisms.

## 2. Materials and Methods

### 2.1. Preparation of CCM

*Cordyceps militaris* was obtained from the Department of Agriculture (DOA) in Thailand. CCM was prepared at the Department of Agricultural Science, Faculty of Agriculture, Natural Resources and Environment, Naresuan University. In brief, the mycelia were cultivated with modified of potato dextrose agar (MPDA) medium under stable conditions at 22°C for 2 weeks. The resultant culture was transferred to potato dextrose broth (PDB) medium, which was then incubated on rotary shaker at 22°C for 2 weeks before transferring the mycelium to sterilized rice cultured medium that contained white rice and silkworm pupae. The fruiting bodies with the inoculums were kept in 12 : 12 h light-dark at 18°C, 60-70% humidity until the mycelium had transformed into the fruiting bodies primordia. Then, the flasks were maintained at 22°C, 80-90% humidity for 64 days before the fruiting bodies had been reaped and immediately frozen at -20°C until used.

### 2.2. Detection of Cordycepin and Adenosine in CCM with High-Performance Liquid Chromatography (HPLC) Fingerprint Analysis

Cordycepin and adenosine in CCM were determined according to Huang et al. (2009) with some modifications. In brief, the fruiting bodies were dried in hot-air oven (55°C, 48 h). The dried samples were ground using a homogenizer, and 1.0 g of powder was added into 10 ml of methanol : water (50/50, *v*/*v*) and sonicated for 30 min, followed by centrifugation at 9,900 g for 15 min 2 times. The obtained supernatant was then filtered through a 0.45 *μ*m filter membrane before injecting into the HPLC system (Shimadzu, Japan) with a column (Restek, Ultra IBD; 150mm × 4.6mm, 5 *μ*m particle size) set at 35°C. The mobile phase was a mixture of water and methanol (90 : 10; *v*/*v*) with the flow rate at 1 ml/min and a UV-vis detector at 254 nm. Five standard solutions of cordycepin and adenosine (Sigma Chemical, MO, USA) (20 *μ*l) were prepared and injected into the HPLC to create standard calibration curves.

### 2.3. Animals

Eight-week-old Sprague-Dawley rats used in this study were specific-pathogen-free (SPF) grade and were purchased from M-CLEA Bioresource Co., Ltd. (Samut Prakan, Thailand). Procedures involving animal subjects were approved by the Naresuan University Animal Care and Use Committee (NUACUC). All animals were handled in accordance with the Guidelines for the Care and Use of Laboratory Animals (National Research Council of Thailand) with an effort to minimize animal suffering. Rats were maintained under controlled temperature (22 ± 1°C) and relative humidity (55 ± 10%) with 12 : 12 hours of reverse light and dark cycle at Naresuan University Centre of Animal Research (NUCAR), which has been accredited by AAALACi. All rats were fed ad libitum a standard diet (CP No. 082; C.P. Company, Bangkok, Thailand) and allowed free access to reverse osmosis (RO) water.

In order to induce diabetes, fifty male rats received a single intraperitoneal injection of STZ (60 mg/kg BW, i.p.) (Sigma-Aldrich, USA), which was dissolved in citrate acid buffer (pH 4.5). Ten male rats in the normal control group received only citrate buffer. After 72 h, fasting blood glucose (FBG) levels were checked using glucometer (Accu-Chek Performa, Roche). Rats with FBG levels higher than 200 mg/dl were used and divided into five groups: (I) DM control, (II) DM+CCM 0.1 g/kg, (III) DM+CCM 0.5 g/kg, (IV) DM+CCM 1.0 g/kg, and (V) DM+sildenafil 5 mg/kg. CCM were weighted and blended thoroughly with a blender and were given by daily oral gavage for 3 weeks. Sildenafil citrate was given only one time 30 min before mating behaviour test.

### 2.4. Surgical Procedure: Ovariectomy

For prevention of pregnancy, female rats were subjected to bilateral oophorectomy surgery before beginning the sexual function assessment. Each adult female rat was anaesthetized by 1.5-2.0% isoflurane (Piramal Critical Cares, Inc., USA) combined with oxygen. The lower abdominal skin and muscle were opened vertically 1 cm, and the uterine horn was pulled out and ligated before removal of the ovary, one at a time. The uterine horn was returned to the peritoneal cavity, and the wound was closed in two layers (abdominal muscle and skin) using sterile sutures. The skin was then disinfected with povidone iodine and covered with Fixomull Stretch®. Each rat received an intramuscular tramadol (5 mg/kg) to ameliorate postoperative pain and allowed at least two weeks for full recovery. The ovariectomized female rats were artificially brought into oestrus phase by the administration of estradiol (0.025 mg/kg) and progesterone (1 mg/kg) at 48 and 4 hours before mating, respectively.

### 2.5. Mating Behaviour Assessment

Mating tests were conducted in a custom made clear glass chamber 50 × 35 × 35cm. Each male rat was allowed to habituate in the chamber for 5 min before introducing a sexually receptive female rat into the chamber. The following male sexual behaviour parameters were calculated after monitoring for 30 min:

- Mount latency (ML). The time interval between the introduction of the female and the first mount by the male
- Intromission latency (IL). The time interval between the introduction of the female and the intromission by the male
- Ejaculatory latency (EL). The time interval between the first intromission and ejaculation
- Mount frequency (MF). The number of mounts from the time of introduction of the female until ejaculation
- Intromission frequency (IF). The number of intromissions from the time of introduction of the female until ejaculation
- Ejaculation frequency (EF). The number of ejaculations in a sexual cycle.

Their behaviour was recorded with the digital VDO camera (LYD-808C, China) for offline analysis by two observers to ensure accuracy.

### 2.6. *In Vivo* Assessment of Erectile Function

After completion of mating test, each male rat was anaesthetized with 2-2.5% isoflurane combination with oxygen. The ventilation rate, pulse rate, temperature, and heart rate were monitored via PhysioSuite® (Kent Scientific, USA). The carotid artery of rat was cannulated to measure mean arterial blood pressure (MAP) by using PowerLab® (AD Instruments, Australia). The penile skin was removed, and a polyethylene tube was inserted with heparinized saline via a 22*-*gauge needle for measuring intracavernosal pressure (ICP). The lower abdomen was opened exposing the cavernous nerve, which was then stimulated via a copper bipolar electrode connected to the PowerLab®. The cavernous nerve was stimulated by electrostimulation, starting from 0.25, 0.50, 0.75, 1, 2, 3, 4, and 5 to 10 volts at a frequency of 20 Hz for 60 sec for each voltage. The results were recorded by LabChart (version 7.3.7; AD Instruments, Australia) connected to a computer. Since electrostimulation slightly lowered MAP, ICP was normalized by MAP as ICP/MAP. Upon completion of experiment, rats were euthanized, and their penis and left testis were immediately collected and stored at -80°C for further analysis. The relative weights of penis and testis were calculated by the following formula: (weightofpenisorlefttestis/bodyweight) × 100.

### 2.7. Determination of Serum Testosterone and Sperm Concentration and Motility

Blood was collected from abdominal aorta and put into the nonanticoagulated tube and stored at 4°C before sending to the Biolab Medical Clinic, Phitsanulok, Thailand, for testosterone analysis. Semen was collected from the caudal epididymis and vas deferens and diluted in 1 M phosphate buffer saline (PBS) at 37°C. Then, 10 *μ*l of sample was transferred into the Makler chamber and analysed under a light microscope. Sperm motility was recorded as video files for offline analysis. The number of sperm was counted and expressed as × 10^6^ per milliliter according to the WHO manual.

### 2.8. Measurement of Penile Nitric Oxide Synthase (NOS) Activity

NOS activity in the penis was determined using the nitric oxide synthase assay kit (Cat. No 482702; Calbiochem®, Germany). The penile tissue was weighted and homogenized with ice-cold 1 M PBS (pH 7.4) by homogenizer (Ultra-turexT8, Germany). The sample was centrifuged at 10,000 g for 20 minutes and filtered through 0.45 *μ*m membrane filter, and then the supernatant was obtained by centrifuging the sample at 100,000 g, 4°C for 15 min. The penile NOS activity was measured, following the manufacturer's instructions. The absorbance at 540 nm was determined using a microplate reader (1401, LabSystem, Finland). Blank wells were used to normalize the yield.

### 2.9. Measurement of Superoxide Dismutase (SOD) Activity

Testicular SOD was measured using the superoxide dismutase assay kit (Calbiochem®, cat #574601, Merck Millipore). Testis tissue was homogenized with buffer (10% *w*/*v*), pH 7.2 (containing 1 mM EGTA, 70 mM sucrose, 20 mM HEPES, and 210 mM mannitol). The homogenate was centrifuged at 1,500 g for 5 min. The supernatant was collected and centrifuged at 10,000 g, 4°C for 15 min. The SOD activity in the sample was then processed according to the manufacturer's instructions and determined by a microplate reader with the absorbance at 450 nm.

### 2.10. Measurement of Thiobarbituric Acid Reactive Substances (TBARS)

Concentrations of TBARS in testes tissues were measured according to the method of Ohkawa et al. [18] with some modifications. Testicular tissue was homogenized (10% *w*/*v*), in ice-cold 1 M phosphate buffer (pH 7.4). The homogenate was centrifuged at 4,000 g, 4°C for 15 min. Sample supernatant (100 *μ*l) was added into the vial containing 1,500 *μ*l of 20% acetic acid (pH 3.5), 200 *μ*l of 8.1% sodium dodecyl sulphate (SDS), and 1,500 *μ*l of 0.8% of thiobarbituric acid (TBA). The mixture was incubated for 60 min at 95°C and immediately cooled on ice, followed by centrifugation at 10,000 g for 3 min. The resulting supernatant was then used as the enzyme source for the determination of the malondialdehyde (MDA) level by the optical density (OD) measurement of the pink complex at 532 nm. MDA values were calculated using tetramethylpiperidine (TMP) as a standard curve and expressed as nmol/mg of protein that determined absorbance at a wavelength of 562 nm. Protein level was measured using the Pierce™ BCA Protein Assay Kit (Thermo Fisher Scientific, USA).

### 2.11. Histological Examination of Testicular Tissues

The right testis was fixed in 10% neutral buffered formalin over 24 hours. The tissues were then processed with 70-100% ethanol and xylene, respectively. The infiltrated tissues were embedded in paraffin blocks and cut at 2 *μ*m by using semiautomatic microtome (Leica, Germany). All slides were stained with hematoxylin and eosin (H&E), and histological changes were observed by light microscopy.

### 2.12. Statistical Analysis

The value of mating parameters, sperm concentration, NOS and SOD activities, and MDA level were presented as mean ± SEM. ICP, MAP, and testosterone levels were presented as mean ± SD. Data were analysed using one-way analysis of variance (ANOVA) followed by Dunn's post-hoc Multiple Comparison Test (Graph Pad Prism 7.4, GraphPad Software, San Diego, USA). *P* values of less than 0.05 were regarded as statistically significant.

## 3. Results and Discussion

### 3.1. Cordycepin and Adenosine Contents in CCM

**Figure 1:**
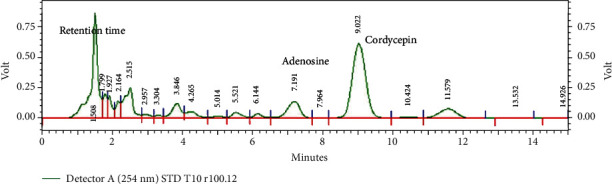
*The* HPLC-UV profile of CCM. The presence of adenosine (Rt = 7.191min) and cordycepin (Rt = 9.022min) was evidenced at 254 nm wavelength.

Many species of Cordyceps are being cultivated in artificial medium for their medicinal and pharmaceutical properties. Cordycepin, a derivative of the nucleoside adenosine, has been shown to be the first and is the main active constituent isolated from *Cordyceps* sp. [9]. In this study, we performed an analysis of bioactive compounds and found that the adenosine and cordycepin contents in CCM were 1,166.59 ± 22.89mg/kg and 4,799.32 ± 9.22mg/kg, respectively (Figure 1). This amount of cordycepin is relatively similar to that cultured in silk worm pupae medium (4.17 ± 1.66mg/g) and higher than that obtained in brown rice medium (2.98 ± 1.41mg/g), as reported by Kang et al. [19]. However, cordycepin production could yield with a maximum of about 445 mg/l, when CM was cultured in submerged conditions [20].

### 3.2. Effect of CCM on Fasting Blood Glucose (FBG) and Reproductive Organ Weight

The antidiabetic activity of CM was demonstrated in type 2 diabetic animal models [13, 14, 21]. In this study, the animal model of type 1 DM was induced by intraperitoneal STZ injection and selectively destroyed pancreatic *β*-cells, inducing an impairment of insulin secretion. Further development of polydipsia, polyuria, and weight loss confirmed diabetes. The body weight change of diabetic rats was lower than those in normal rats (Table 1). FBG levels of DM control rats were significantly higher than those in normal rats. In our results, treatment of CCM (0.1, 0.5, and 1.0 g/kg BW) did lower FBG but not significantly different. The greater baseline FBG levels in diabetic rats of our study may have contributed to the less decrease in FBG levels with CCM treatment.

Relative testis weight weight were not different among groups except the DM and DM+Sildenafil groups, which showed a significant decrease when compared with control rats. This reduction in testis weight of diabetic rat is consistent with the report showing the negative effects of STZ-induced diabetes on the testes weight and testicular lesion in adult albino rats [22].

### 3.3. Effect of CCM on Mating Behaviour

Many studies have reported diabetes can lead to reduce sexual motivation. In the present study, the results of male mating behaviour showed that all males displayed mounts and intromissions and all females responded with lordosis to every mount received under experimental conditions. Diabetes induced a significant reduction in sexual motivation and mating behaviour. As shown in Table 2, decreased sexual activity was found in diabetic rats as a decrease in MF, IF, and EF and increase in ML and IL were observed in these rats. This observation was consistent with the study of Escrig [23] and Minaz [24]. Treatment with CCM (0.1 and 0.5 mg/kg) or sildenafil restored the sexual function in diabetic rats, as evident by an increase in IF and a decrease in ML and IL compared to DM control rats. However, the result of EF in diabetic rats treated with CCM 1.0 g/kg which was not different from DM control remained equivocal. In view of this, CCM treatment could be beneficial in reducing the deleterious effect of diabetes on sexual functions, and sildenafil can also enhance sexual motivation and function in diabetic rats.

### 3.4. Effect of CCM on ICP

**Figure 2:**
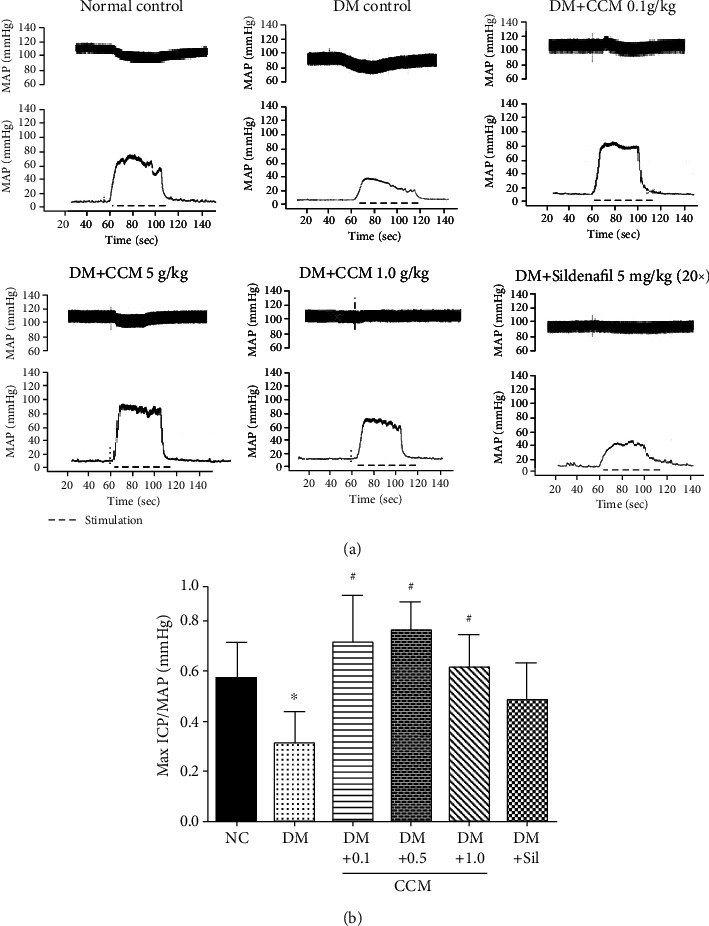
(**a**) Representative ICP and MAP tracings at 5 voltages in the six groups. (**b**) Bar graph depicting Max ICP/MAP ratio. Values represent mean ± SD(*N* = 10). ∗*P* < 0.05 compared with the normal control group, ^#^*P* < 0.05 compared with the DM control group.

Erection function in rodents can be evaluated by measuring ICP [25]. The magnitude of erectile activity was quantified as the ratio of ICP to MAP, which is the common functional index to determine erectile function. Following electrical stimulation of cavernous nerve at 1-10 V, it was found that electrical stimulation increased ICP in a voltage-dependent manner.  Figure 2(a) illustrates the quantitative measurement of ICP and MAP during electrical stimulation of the cavernous nerve at the voltage of 5 V for 60 s. The DM control group revealed a significant decrease in ICP and ICP/MAP ratio compared with the normal control rats (Figure 2(b)). The results demonstrated that treatment with CCM (0.1, 0.5, 1.0 g/kg BW) significantly improved the ICP/MAP ratio in diabetic rats. Additionally, a marked increase in ICP/MAP ratio was observed in diabetic rats that received sildenafil. It is important to note that sildenafil was given one time before mating behaviour test. Our results are in support with previously published report, which revealed that the magnitude of the increase in ICP after cavernosal nerve stimulation was significantly lower in the STZ-diabetic rats, and a significant increase in peak ICP at the 5 and 7.5 V settings was observed in STZ-diabetic rats administered sildenafil [26].

### 3.5. Effect of CCM on Sperm, Serum Testosterone, and Testicular Tissue

**Figure 3:**
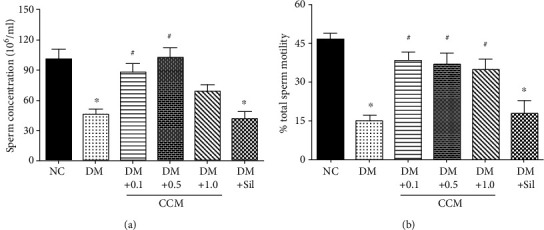
Effect of CCM on sperm function, serum testosterone, and penile nitric oxide synthase activity after 3-week administration. (**a**) Sperm concentration in epididymis and vas deference. (**b**) Percentage of total sperm motility in epididymis and vas deference. Values represent mean ± SD(*N* = 10/group*).*∗*P* < 0.05 compared with the normal control group, and ^#^*P* < 0.05 compared with the DM control group.

DM is known to cause many systemic complications including male infertility [27]. As observed in this study, the male sperm counts and sperm motility significantly declined in diabetic rats as compared to the normal control rats. Treatment of CCM (0.1 and 0.5 g/kg) significantly increased the number of sperm, as well as sperm motility in diabetic rats (Figure 3). Our findings are in agreement with previous studies reporting CM improved sperm quality and quantity in rats [15], and long-term administration of cordycepin has been shown to counteract the decline of testicular function in middle-aged rats [16]. A significant improvement in sperm quality has also been reported in aged rats that received cordycepin extracted from CM [28]. In contrast, the number of sperm and its motility were relatively unchanged in diabetic rats treated with sildenafil citrate. This drug seemed to consistently demonstrate an unusual side effect on the histological structure of the testis of adult albino rats when given for a long time [29].

**Figure 4:**
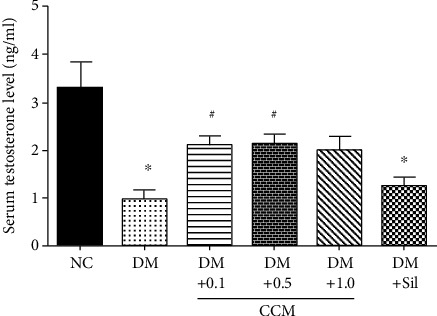
Effect of CCM on serum testosterone level of male rats. Values represent mean ± SD (*N* = 10). ∗*P* < 0.05 compared with the normal control group, and ^#^*P* < 0.05 compared with the DM control group.

Testosterone level is an important indicator of male reproductive health. As might be expected, serum testosterone levels were markedly lower in diabetic rats than in normal control rats (Figure 4). Administration of CCM (0.1 and 0.5 g/kg) and sildenafil significantly increased serum testosterone in diabetic rats. Testosterone level in the DM+CCM 1 g/kg showed a marked increase, but the difference from the DM control did not reach statistical significance. In agreement with this, an increase in testosterone production was observed in the SD rats that received fruiting bodies of CM cultured on bee drone and brown rice medium for 4 weeks [30]. Undeniably, the increasing serum testosterone level could be induced by cordycepin according to Hsu's report [31]. Treatment of sildenafil has also been shown to increase testosterone levels with improvement of erection power in either men with ED [32] or type 2 diabetic men with ED [33].

**Figure 5:**
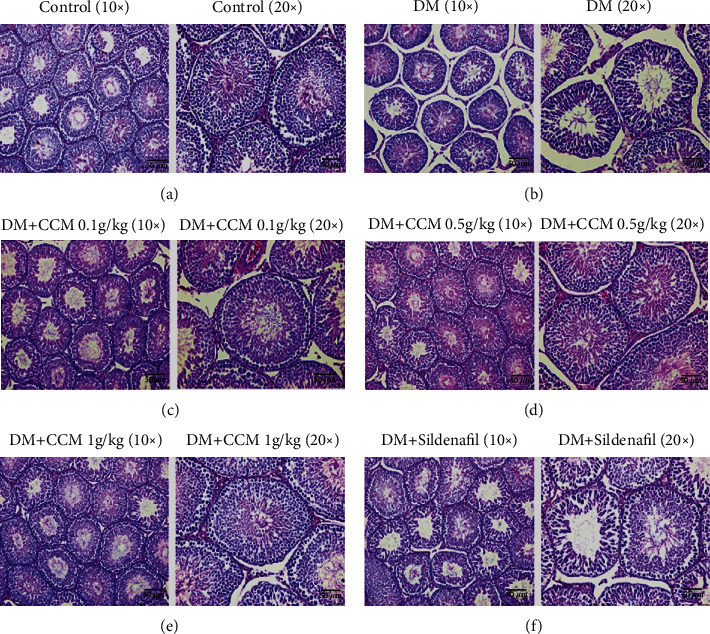
Example photomicrographs of rat testis stained with hematoxylin and eosin (H&E*).* The photomicrographs showed the magnification: × 10 (left) and × 20 (right) in each group*. ( ***a**) Normal control group. (**b**) DM control group. (**c**) DM+CCM 0.1 g/kg group. (**d**) DM+CCM 0.5 g/kg group. (**e**) DM+CCM 1 g/kg group. (**f**) DM+Sildenafil 5 mg/kg group.

The testicular histology of normal control rat showed a normal testicular structure, normal outline of the seminiferous tubules, and all levels of spermatogenic cells (Figure 5(a)). In agreement with the results of previous studies, our study showed abnormal histology of seminiferous epithelium and the size of seminiferous tubules in the DM control group (Figure 5(b)). Additionally, the cellular levels of spermatocytes and spermatids were reduced, and the connections between cells were disappeared in these rats. It was evident that the degree of spermatogenetic impairments in diabetic rats correlated with the reduction in testis weight (Table 1) and the testosterone levels (Figure 4). Interestingly, CCM-treated diabetic rats showed less severe of morphologic alterations (Figures 5(c)–5(e)). This result is in agreement with the study of Sohn [16] who reported the positive effect of cordycepin in spermatogenesis-related parameters in middle-aged rats. Beneficial effects of cordycepin were also reported in age-associated alterations in testicular function [28].

### 3.6. Effect of CCM on NOS Activity in Penile Tissue

**Figure 6:**
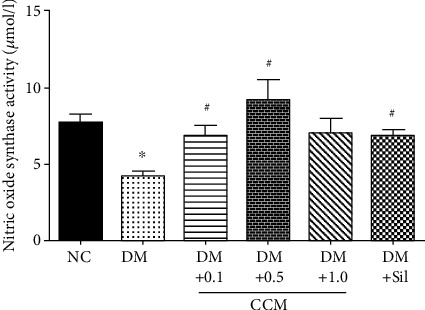
Effect of CCM on NOS activity in penile tissues. Values represent mean ± SEM(N = 6/group*).*∗*P* < 0.05 compared with the normal control group, and ^#^*P* < 0.05 compared with the DM control group.

Nitric oxide (NO), produced either by nerves or by endothelium, plays an important role in the relaxation of corpus cavernosum smooth muscle and vasculature [34]. In this study, nitric oxide synthase (NOS) activity was measured from penile tissue after completion of ICP measurement (Figure 6). A statistically significant decrease in penile NOS activity was observed in diabetic rats compared to the normal control group. The result of NOS measured from penile tissue of diabetic rats in DM+CCM (0.1 and 0.5 g/kg) significantly increased, while increased level of NOS in the DM+CCM 1 g/kg was not evident. The increase in max ICP/MAP and NOS levels was observed in this study, which might be due to the cordycepin, which is analogues of adenosine, and has been shown to stimulate in vitro and in vivo steroidogenesis in mouse Leydig cells through the activation, at least, of the protein kinase A pathway [35]. Likewise, the study of Tostes [36] indicated that adenosine may contribute to penile erection through the activation of adenosine receptors, A_2A_ and A_2B_ receptors, as observed in mouse corpus cavernosum. Accumulating evidence indicates the role of adenosine in penile erection as reviewed by Phatarpekar [37].

### 3.7. Effect of CCM on Testicular SOD Activity and MDA Levels

**Figure 7:**
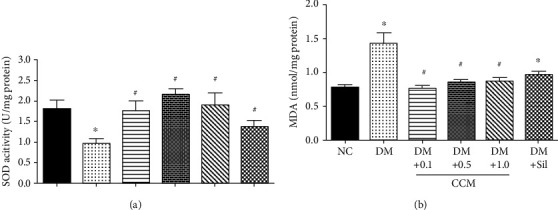
Effect of CCM on testicular SOD and MDA after 3-week administration. (**a**) SOD activity. (**b**) MDA level. Values represent mean ± SD(N = 10/group*).*∗*P* < 0.05 compared with *the* normal control group, and ^#^*P* < 0.05 compared with the DM control group.

SOD is a necessary antioxidant enzyme which maintains body reactive oxygen species (ROS). In this study, SOD levels were measured from the testicular tissues, and the result of disclosed SOD activity in the DM control group was significantly reduced when compared with the normal control group. Interestingly, the SOD levels in the DM+CCM (0.1, 0.5, and 1 g/kg) and DM+Sildenafil groups were significantly augmented when compared with the DM group (Figure 7(a)). On the other hand, testicular MDA levels showed significant elevation in DM control compared with normal control group. Treatment with CCM and sildenafil significantly diminished the elevated levels of testicular MDA (Figure 7(b)). An elevated testis MDA level that correlated with decreased SOD activity in DM control rats was observed in this study, and consistent with the study of Aybek [38], which reported an elevation in MDA level and a decrease in SOD level in the diabetic groups. Since CCM administration increased the SOD activity while decreased MDA levels in the testicular tissue, it seems that the treatment with CCM moderates testicular tissue damage in diabetic rats possibly through its antioxidant properties [8, 39].

## 4. Conclusions

One of the reproductive complications of DM in men is erectile dysfunction. In this study, diabetes had a negative effect on sexual function, penile erection, sexual organ weight, and sperm parameters with a parallel decrease in the level of testosterone of male rats. The diabetic rats that received CCM showed a significant improvement in mating behaviour, erection function, and testicular function as observed by increasing ejaculation frequency, ICP/MAP ratio, and testosterone level, respectively. These improvements may be due at least in part to bioactive compounds that could improve testosterone production and its antioxidant properties. Additionally, cordycepin itself acts as adenosine analogue mediating the vasodilation to improve erectile function. Based on our findings, cultured *C. militaris* could be used as herbal drug for ameliorating reproductive dysfunctions secondary to DM.

## Figures and Tables

**Table 1:** Fasting blood glucose (mg/dl) and relative organ weight *(%* BW).

Groups	FGB (mg/dl)		Organ weight (%BW)		BW change (g)
	Initial	Final	Penis	Testis	
Normal control	111.6 ± 3.25	105.0 ± 3.82	0.449 ± 0.02	1.781 ± 0.06	77.07
DM control	110.2 ± 3.00	528.0 ± 20.15∗	0.413 ± 0.02	1.587 ± 0.03∗	-28.33
DM+CCM 0.1	104.2 ± 4.25	447.4 ± 38.66	0.381 ± 0.02	1.620 ± 0.03	10.70
DM+CCM 0.5	105.4 ± 3.69	448.1 ± 36.06	0.442 ± 0.03	1.711 ± 0.02	33.60
DM+CCM 1.0	102.6 ± 3.24	424.8 ± 30.76	0.332 ± 0.01	1.650 ± 0.05	-6.00
DM+Sildenafil	109.3 ± 2.82	522.6 ± 17.44	0.401 ± 0.02	1.591 ± 0.03∗	-13.5

Note: values represent mean ± SD, (*N* =10). ∗*P* < 0.05 different from normal control group.

**Table 2:** Effects of 3-week administration of vehicle, CCM (0.1, 0.5, and 1.0 g/kg BW) or sildenafil citrate (5 mg/kg BW) on mating behaviour in STZ-induced diabetic rats.

Group	Parameters of mating behaviour					
	Mount latency (ML; min)	Intromission latency (IL; min)	Ejaculation latency (EL; min)	Mount frequency (MF; count)	Intromission frequency (IF; count)	Ejaculation frequency (EF; count)
Normal control	0.29 ± 0.10	1.93 ± 0.76	18.46 ± 2.36	24.20 ± 5.56	57.80 ± 6.40	1.70 ± 0.26
DM control	0.53 ± 0.04∗	12.69 ± 3.72∗	25.91 ± 2.02	21.35 ± 3.20	20.60 ± 7.59∗	0.50 ± 0.22∗
DM+CCM 0.1	0.24 ± 0.04^#^	0.67 ± 0.15^#^	17.52 ± 2.42	21.55 ± 3.09	51.00 ± 4.32	2.00 ± 0.33^#^
DM+CCM 0.5	0.22 ± 0.05^#^	0.80 ± 0.24^#^	17.50 ± 2.00	13.10 ± 2.92	57.25 ± 2.94^#^	2.00 ± 0.33^#^
DM+CCM 1.0	0.29 ± 0.07^#^	6.59 ± 3.71	24.37 ± 2.39	24.55 ± 7.11	35.60 ± 9.24	0.60 ± 0.27∗
DM+Sildenafil	0.14 ± 0.02^#^	8.62 ± 3.94	18.98 ± 3.24	15.38 ± 3.06	23.30 ± 5.86∗	1.10 ± 0.32

Note: values represent mean ± SD (*N* = 10). ∗*P* < 0.05 different from the normal control group, and ^#^*P* < 0.05 different from the DM control group.

## Data Availability

The behavioural data used to support the findings of this study are included within the article.

## Abbreviations

A2A: Adenosine receptor type 2A
A2B: Adenosine receptor type 2B
AAALACi: Association for the Assessment and Accreditation of Laboratory Animal Care International
BCA: Bicinchoninic acid
BW: Body weight
CCM: Cultured cordyceps militaris
CM: *Cordyceps militaris*
DIED: Diabetes-induced erectile dysfunction
DM: Diabetes mellitus
DOA: Department of Agriculture
EF: Ejaculation frequency
EL: Ejaculation latency
ED: Erectile dysfunction
FBG: Fasting blood glucose
HPLC: High-performance liquid chromatography
ICP: Intracavernosal pressure
i.p.: Intraperitoneal injection
IF: Intromission frequency
IL: Intromission latency
MAP: Mean arterial pressure
MDA: Malondialdehyde
MF: Mount frequency
ML: Mount latency
MPDA: Modification of potato dextrose agar
NO: Nitric oxide
NOS: Nitric oxide synthase
NUACUC: Naresuan University Animal Care and Use Committee
NUCAR: Naresuan University Centre for Animal Research
PDB: Potato dextrose broth
RO: Reverse osmosis
SD: Sprague-Dawley
SOD: Super oxide dismutase
SPF: Specific-pathogen-free
STZ: Streptozotocin
TBARS: Thiobarbituric acid reactive substance
WHO: World Health Organization.
